# Spray Congealing: An Emerging Technology to Prepare Solid Dispersions with Enhanced Oral Bioavailability of Poorly Water Soluble Drugs

**DOI:** 10.3390/molecules24193471

**Published:** 2019-09-25

**Authors:** Serena Bertoni, Beatrice Albertini, Nadia Passerini

**Affiliations:** Department of Pharmacy and BioTechnology, University of Bologna, Via S. Donato 19/2, 40127 Bologna, Italy; serena.bertoni4@unibo.it (S.B.); beatrice.albertini@unibo.it (B.A.)

**Keywords:** spray chilling, spray cooling, microparticles, solubility enhancement, dissolution rate, polyethylene glycol, poloxamer, Gelucire^®^

## Abstract

The low and variable oral bioavailability of poorly water soluble drugs remains a major concern for the pharmaceutical industry. Spray congealing is an emerging technology for the production of solid dispersion to enhance the bioavailability of poorly soluble drugs by using low-melting hydrophilic excipients. The main advantages are the absence of solvents and the possibility to obtain spherical free-flowing microparticles (MPs) by a relatively inexpensive, simple, and one-step process. This review aims to fully describe the composition, structure, physico-chemical properties, and characterization techniques of spray congealed-formulations. Moreover, the influence of these properties on the MPs performance in terms of solubility and dissolution enhancement are examined. Following, an overview of the different spray congealed systems developed to increase the oral drug bioavailability is provided, with a focus on the mechanisms underpinning the bioavailability enhancement. Finally, this work gives specific insights on the main factors to be considered for the rational formulation, manufacturing, and characterization of spray congealed solid dispersions.

## 1. Introduction

Oral route is the most common way of drug administration because of its simplicity and ease of administration. About 80% of the dosage forms in the worldwide market are administered orally [1]. Compared to oral liquid medicines (e.g., oral solution or suspensions), solid oral dosage forms have many benefits for the patient, such as convenience and simplicity of use, accurate dosing of the active ingredient, and no need of preparation/manipulation of the medicine before administration. From the industrial perspective, solid forms also offer the advantages of good stability and generally moderate production costs. For these reasons, most of the new chemical entities (NCEs) under development are intended to be marketed as solid dosage forms for oral administration [2]. Because of the recent advances in combinatory chemistry and high-throughput screening methods, nowadays the NCEs are frequently large-molecular-weight lipophilic compounds. Therefore, poorly water soluble drugs constitute the 70% of the new drug candidates [3]. Various physical-chemical properties contribute to make a compound poorly soluble and those include its complex structure, size, high molecular weight, high lipophilicity (log P), compound H-bonding to solvent, intramolecular H-bonding, intermolecular H-bonding (crystal packing), polymorphic forms, ionic charge status, pKa, and salt form [4]. According to the Biopharmaceutical Classification System (BCS), a drug compound is poorly soluble if the highest dose strength is not soluble in 250 ml aqueous media over a pH range of 1–7.5 at 37.5 °C [5]. Thus, poor aqueous solubility represents a serious concern if the clinical dose of drug cannot dissolve in the available volume of gastrointestinal fluids [6]. 

Different strategies have been proposed over the years to address the issue of poorly water soluble drugs and they include particle size reduction, formation of nanocrystals and co-crystals, pH adjustment, use of cosolvents, self-emulsifying drug delivery systems (SEDDS), inclusion complexes, nanosuspensions, and solid dispersions. Among these methods, the development of solid dispersion (SD) has demonstrated to be one of the most promising approaches [7]. The term SD refers to a system in which the drug is dispersed in a solid inert matrix and it consists of at least two different components which are commonly a hydrophilic matrix and a hydrophobic drug [8]. Generally, SD can enhance oral bioavailability by allowing a rapid release of the drug into the solution in a supersaturated state above its equilibrium solubility and maintaining this state for as long as possible [9]. 

Numerous technologies can be used for the manufacturing of SD. The methods for the preparation of SD are classified in solvent-based methods, melting-based methods, and mechanochemical activation. All kinds of high energy milling are considered as mechanochemical activation [10]: high levels of mechanical energy may result in the crystal lattice disruption of materials, causing polymorphic transformations, crystal defect generation, amorphization, and other additional physical and chemical changes in a crystalline drug [11]. Spray-drying represents the main widespread solvent-based method to obtain SD; it involves the evaporation of a solvent used to solubilize the SD components, with the formation of solid particles. Spray drying is a versatile technique to make SD. In addition, it is widely used to prepare microparticles, nanoparticles, self-emulsifying delivery systems, eutectic mixtures, and may use either organic or inorganic polymeric carriers [12]. The main disadvantages are the relatively high process temperature, use of solvents, small particle size distribution, and high maintenance cost. Melting-based methods involve either the melting of the carrier or most frequently the softening of the thermoplastic polymeric carrier. The key feature of the melting-based technologies are the absence of solvent, either aqueous or organic, with related advantages of no toxicity related to the presence of organic solvents and the possibility to be applied for the loading of hygroscopic and water-sensitive drugs. Hot melt extrusion is the most established melting-based technique and it has been extensively explored for the manufacturing of SD [13]. Beside to the numerous advantages, hot melt extrusion presents some drawbacks, such as the need of downstream processes to obtain the final product. Spray congealing (SC) has been recently proposed as an emerging technology for the manufacturing of SD in form of microparticles (MPs).

The aim of this review is to illustrate the potential of SC in the preparation of MPs aiming to enhance the oral bioavailability of poorly water soluble drugs. To this purpose, different examples of applications of this technology for bioavailability enhancement are discussed. The composition, properties and characterization techniques of spray congealed-formulations are described and their influence on the MPs performance in terms of solubility and dissolution enhancement are fully examined. Finally, the review aims to provide specific insights on the main factors to be considered for the rational formulation, manufacturing, and characterization of spray congealed-SD.

## 2. Spray Congealing Technology: General Aspects

**Figure 1:**
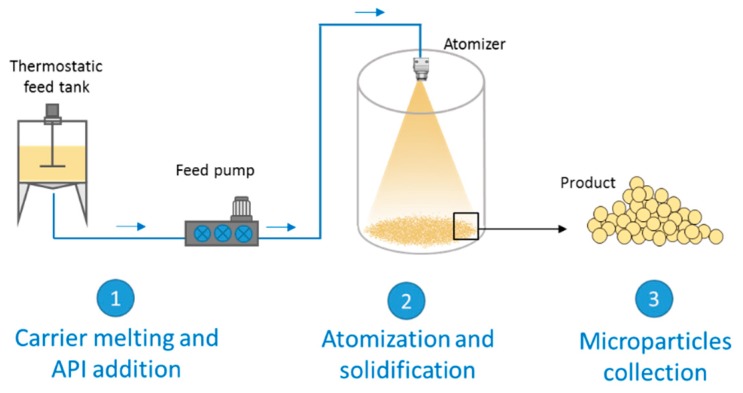
Scheme of a general spray congealing apparatus with the different steps for the production of microparticles.

In the past decades SC, also called spray chilling or spray cooling, has attracted increasing attention because it is a simple technique, less time and energy consuming compared to other methods to obtain SD [14,15]. Other advantages include the ability to obtain spherical free-flowing MPs suitable for tableting or capsule filling without the need of other downstream processes (e.g., secondary drying, milling, granulation) and high encapsulation efficiency values (90–100%). From the industrial perspective, SC is easily scalable and can be adapted to the “continuous manufacturing”. As regards the equipment and mechanism of atomization, SC is closely associated with spray drying. Generally, spray drier apparatus can be used for SC processes with modifications. Although both techniques are based on the generation of droplets through atomization of a fluid, the mechanisms of MPs formation in spray drying and spray congealing are basically different, as centered on the evaporation of solvent and on the hardening of a molten material, respectively. Essentially, in the spray drying process a liquid solution (or suspension) is converted to a powdered solid by pumping the liquid feed into a drying chamber via a nozzle where fine droplets rapidly encounter a hot drying gas. The wet gas and dry particles are then separated by a cyclone and/or bag filter and the powdered product is dispensed into a collection vessel and/or bag filter [12]. On the other hand, SC is based on the atomization of a fluid (solution or a suspension of a drug in a melted carrier) into a chamber maintained at a temperature below the carrier melting point. The steps of the process are schematized in Figure 1. The first step involves the preparation of the fluid consisting of the molten carrier, kept at a temperature above its melting point, and the active ingredient. Excipients suitable for SC are substances with melting temperatures generally ranging from 35 to 90 °C. In the second step, the molten fluid stream is broken into small droplets by means of an atomizer. Subsequently the molten droplets solidify upon cooling in the chamber, producing the final solid spherical MPs. The performance of the spray congealing process strictly depends on the atomization efficiency of the molten fluid. Thus, the most important step is the atomization phase, which can be performed using various type of atomizers: pressure, two-fluids (also called pneumatic or air nozzles), rotary and ultrasonic nozzles. The reader interested in details on the different equipment and nozzle utilized in the spray congealing is referred to a recent review, where they are presented and fully discussed [16]. 

SC has been used in a wide range of applications, including food and pharmaceutical area. The applications of SC in the production of different oral and non-oral drug delivery systems have been described in a recent review [15]. Specifically, the pharmaceutical applications of this technology cover a wide area, including modified release systems, taste masking, bioavailability enhancement, API protection from different causes of degradation, and encapsulation of biologic drugs.

Spray congealed MPs are a multiparticulate dosage form as they consist of a multiplicity of small discrete units, each containing a fraction of the total amount of the administered API. Multiparticulate oral drug delivery systems are preferred over single unit dosage forms because of their comparatively greater dispersibility in the GIT, reduced-risk of dose dumping and systemic toxicity, decreased dosing frequency, increased patient compliance, lesser variability in concern to absorption, and accurate dosing [17]. Moreover, because of the small size, multiparticulates are less dependent on gastric emptying, resulting in less inter and intra-subject variability in gastrointestinal transit time. Above all, they represent a flexible dosage form and, therefore, they are particularly attractive for developing patient-tailored pharmaceutical products.

## 3. Structure and Composition of Spray Congealed SD 

Spray congealed SD for bioavailability enhancement of poorly water soluble drug can be considered SD in form of hydrophilic MPs, consisting in spherical particles with a size in the range of 10 μm–500 μm. More specifically, the spray congealed product consists on microspheres. Opposite to microcapsules, where the API is confined within the microcapsule by a surrounding layer of excipient, spray-congealed microspheres are a matrix system where the active compound is evenly distributed throughout the carrier. In particular, the carriers employed in SC for bioavailability enhancement application include hydrophilic low-melting materials such as polyethylene glycol (PEG) and its surface active derivatives poloxamers and polyoxylglycerides (commercially known as Gelucires^®^). They are all semicrystalline carriers, as they contain both crystalline and amorphous domains [18,19,20]. 

**Figure 2:**
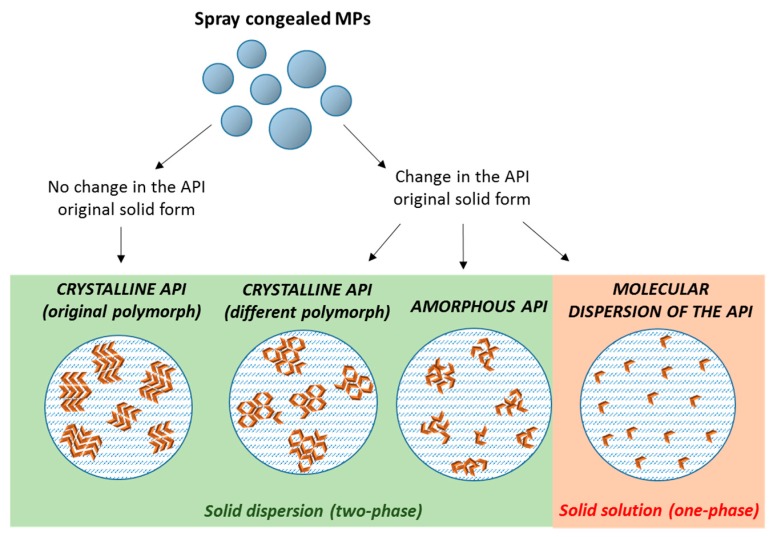
Scheme depicting the different type of solid dispersions that can be obtained by spray congealing (orange arrows represent individual API molecules).

The dispersion of poorly water soluble APIs in these carriers by SC can result in the formation of either *solid dispersions* with crystalline or amorphous drug molecules, or *solid solutions* of the drug molecularly dispersed within the carrier. Often, an intermediate situation is likely to occur, especially at high drug contents, when there is an excess of drug (superior than its solubility in the carrier). Therefore, MPs with the API in different physical states (crystalline, amorphous, or partially crystalline) can be obtained [21], according to the drug and carrier chemico-physical characteristics, their miscibility and potential interactions (Figure 2). The type of the obtained system is strictly dependent on the specific properties of API and carrier (API melting point, API solubility/miscibility in the carrier, API:carrier ratio), and thus it has to be determined on a case-by-case basis.

**Figure 3:**
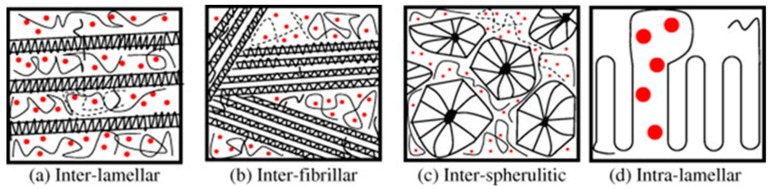
Possible mechanisms of segregation in drug/polyethylene oxide solid dispersion: inter-lamellar (**a**), inter-fibrillar (**b**), inter-spherulitic (**c**) and intra-lamellar (**d**). Red points represent drug molecules. Reproduced with permission from [24].

Among the semicrystalline carriers, PEG has been widely used as a hydrophilic carrier in the preparation of SD formulation, because of its low toxicity and low costs [22]. It is a polymer of ethylene oxide, available in a range of molecular weights ranging from 200 to millions g/mol. The most commonly used for SD are solid PEGs with molecular weight of 1500–6000 g/mol. PEG exhibits a melting point ranging from ca. 55 °C to 65 °C depending of its molecular weight and it is often used as excipient for melting-based processes. Because of its very low Tg values (from −95°C to −17°C) [23], the stabilization of the amorphous form it is extremely difficult and therefore most PEG-based SD are characterized by PEG crystallization during the preparation process or during storage. Recent studies highlight that the API can be located in various domains within the semicrystalline SD and thus multiple microstructures can be obtained (Figure 3), resulting in systems with different properties. For example, aceclofenac and chlorpropamide SD favored the interlamellar incorporation of the drug in the PEG matrix. Haloperidol, a rapidly crystallizing compound, was excluded from the interlamellar region. Loratadine, with a low solubility in PEG, showed more complex phase behavior that depended on the crystallization temperature [20].

Gelucires^®^ are semisolid waxy materials with amphiphilic nature widely used for the manufacturing of spray congealed SD. They are composed of a mixture of mono-, di-, and triglycerides, esters of PEG and free PEG. Gelucires^®^ are characterized by two numbers, the first one referring to the melting point and the second to the theoretical HLB value [25]. Hydrophilic Gelucires^®^ are used as solubilization agents for poorly soluble compounds because of their property of forming a dispersed system in water and water/cosolvent mixtures [26]. The most used compound for spray congealed systems is Gelucire 50/13, a lipid-based excipient with a mean HLB of 11 and a melting temperature around 50 °C [27].

Poloxamers are ABA-type triblock copolymers composed of polyoxyethylene (A) and polyoxypropylene (B) units with high solubilizing capacity. Because of their chemical structure, they are characterized by an amphiphilic nature, which makes them useful non-ionic surfactants that are employed in many industrial fields. Among these, poloxamer 407 (P407), with an hydrophilic–lipophilic balance (HLB) of 22 [28], is the most commonly used for its low toxicity and its compatibility with numerous biomolecules and excipients [29]. They have been used for the production spray congealed MPs [30,31] although their application as unique carrier may be limited by their high viscosity at the molten state.

## 4. Properties and Characterization of Spray Congealed SD

**Figure 4:**
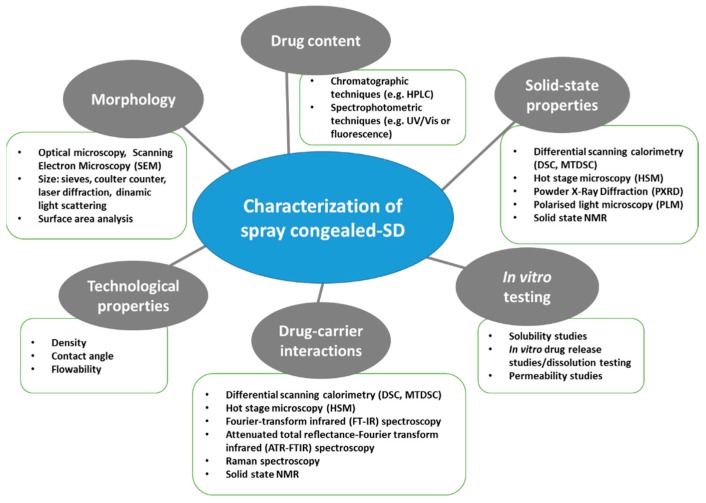
Schematic classification of the most commonly used techniques for the characterization of spray congealed SD.

A thorough evaluation of the spray congealed SD properties is essential as they have a direct impact on the technological and biopharmaceutical behavior of the system. Figure 4 shows a schematic classification of the relevant MPs properties and of the techniques used for their analysis.

### 4.1. Determination of the Drug Content

The actual or experimental amount of API in the MPs following SC process is defined as *drug content* and can be expressed as the mass percentage (% *w/w*). The experimental drug content is strictly related to the drug:carrier ratio used in the preparation of the particles, although it can be influenced by process variables (e.g., operating parameters and equipment) and formulation variables (e.g., drug and carrier characteristics). Generally, the particles are opportunely treated (e.g., with solvents or heating) to dissolve/melt the carrier in order to release the entrapped drug, and the experimental amount of active compound is assayed by means of an appropriate analytical method. The actual drug content can vary according to the MPs size and theoretical drug loading.

The drug content value can be used as a quick indication to estimate the total amount of MPs to be administered, in relation to the therapeutic daily dose of each specific drug. The distinctive property of spray congealed MPs in achieving very high drug content values, even up to 50% *w/w*, is extremely important in view of a clinical application. High drug:carrier ratio of MPs represents one of the most important advantage of these systems, compared for example with nano-sized formulations which generally allow low drug loading, resulting in frequently repeated administration and thereby high cost and increased side effects [32]. 

Although higher drug content values are desirable because of the above-mentioned reasons, a possible negative influence of a high drug-carrier ratio on the biopharmaceutical properties of SD should be considered when the goal is the enhancement of its oral bioavailability. In the case of spray congeled SD of acetazolamide in poloxamer 237, it was observed that different ratios between drug and carrier influenced the solubility enhancement of the SD. Specifically, by decreasing the drug content, the solubility of acetazolamide increased significantly (2.228, 4.010, and 8.286 mg/ml for SD with drug:carrier ratio of 1:1, 1:1.5, and 1:2, respectively). In the same study, the dissolution profiles were also affected by the drug amount, and in particular lower drug contents led to higher improvements in drug dissolution rate. The best dissolution performance was obtained by the SD with the highest percentage of poloxamer 237 [33]. However, poloxamers are known to have thermoreversible gelation properties; before poloxamer dissolution, formation of gel layer might occur in the highly concentrated polymer regions, at the particle-medium interface. Formation of gel layer around the particles causes the retardation of drug release, due to an increase in viscosity, an effect that becomes more pronounced at higher polymer concentrations. Accordingly, the increase in poloxamer ratio over 1:1 in solid dispersions decreased intrinsic dissolution rate of desloratadine SD in poloxamer 188 and 407 [34]. In case of SD containing the poorly water soluble carbamazepine in the same poloxamers, the highest drug release was observed with drug:carrier ratio of 1:1, whereas 1:2 and 1:3 weight ratios led to a less pronounced dissolution enhancement. [35]. Conversely, a study on the bioavailability enhancement of the anthelminthic drug praziquantel (PZQ) from Gelucire-based systems showed that the increase of the PZQ content considerably decreases the dissolution rate of the drug. In particular, all formulations showed a similar improvement in drug dissolution compared to pure drug in the first 20 min, followed by a significantly enhanced dissolution of 5 and 10% PZQ-loaded systems compared to those loaded with 20 and 30% of PZQ [36]. One possible explanation can be found considering that a drug concentration closer to the maximum drug solubility is more easily achieved by the formulations having higher drug amount, thus leading to a plateau in the later phase of the dissolution test. Differently from these two studies, no significant difference was observed on the dissolution of carbamazepine from Gelucire^®^ 50/13-based MPs between different drug contents (5, 10, 15, and 20% *w/w*) [37]. In particular, a previous study also noted that increasing the percentages of Gelucire^®^ in the formulation above 50% *w/w* do not result in a positive effect on the dissolution performance [38]. 

These examples demonstrated that the performance of spray congealed SD, as SD in general, is greatly affected by the drug:carrier ratio. Typically, decreasing carrier concentrations in favor of high drug amount leads to lowered wettability and thus decreased dissolution enhancement. In case of amorphous SD with high drug contents, the risk of possible nucleation and crystallization of the drug from the drug-rich solid phase during dissolution is also enhanced. Moreover, SD with high drug concentration are also less stable during storage at elevated temperatures and relative humidity [39].

### 4.2. Particle Size

**Figure 5:**
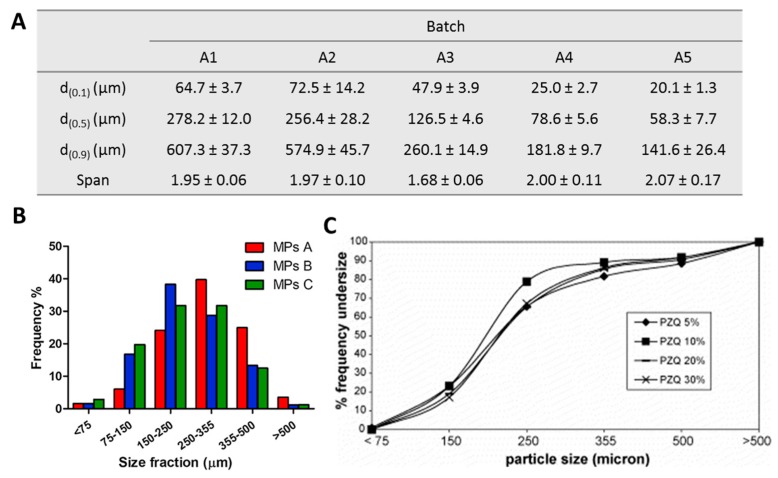
Particle size distributions of spray congealed microparticles (MPs) based on Gelucire^®^ 50/13. (**A**) Effect of different atomizing air pressure (increasing pressure values passing from A1 to A5); (**B**) composed of Gelucire^®^ 50/13 and Gelucire^®^ 48/16 in ratios of 100:0 (MPs A), 50:50 (MPs B), and 30:70 (MPs C) and loaded with 10% *w/w* of indomethacin; (**C**) containing different amount of drug praziquantel (PZQ). Figures adapted with permission from [19,36,45].

The particle size can be expressed in terms of average particle diameter ± standard deviation or as median diameter (d50), namely the particle size below which 50% of the sample (in terms of volume or mass) lies. The d10 and d90 (particle size below which 10 and 90% of the sample lies in terms of volume or mass) are also used [40]. Alternatively, the particle size distribution can be reported, giving more information about the mono- or poly-dispersibility of the MPs batch. MPs with diameters ranging from few micron to hundreds of micron are commonly obtained by SC, as shown in Figure 5. The average size and the size distribution strongly depend on the type of atomizer. Employing two-fluid nozzle, which is the most employed one in SC process, the particles size is influenced by both process and formulation parameters. For example, increasing the atomizing pressure resulted in a decrease of the MPs size (Figure 5A), while the increase of the feeding rate produces inhomogeneous particles distribution. The most important formulation parameter influencing MPs size is the viscosity of the molten fluid. Lower liquid viscosity leads to smaller particles while larger particles are obtained in case of higher liquid viscosity [41]. The viscosity of the molten fluid depends on the carrier type (e.g. Gelucire® 50/13 and Gelucire® 48/16 in different ratios led to different size distribution, Figure 5B), temperature, and addition of solids (APIs or additives). For example, the addition of Aerosil® determined larger particle size by increasing the molten mixture viscosity [42]. On the contrary, using an ultrasonic nozzle, the addition of different amounts of APIs was noticed not to influence the size distribution in case of Gelucire-based MPs containing PZQ (Figure 5C). The particle size is a fundamental property of MPs and profoundly influences the biopharmaceutical performance of the system. In vitro dissolution studies performed on different size fractions of CBZ-loaded MPs showed that dissolution rate tends to increase with smaller MPs [37]. Decrease in MPs size led to the enhancement in drug dissolution rate because the wider surface area of small microspheres compared to bigger ones allows higher contact with the aqueous fluids and therefore faster dissolution [43]. Similar tendency was observed in the dissolution of indomethacin from stearic acid-based MPs containing a SD of the drug with a hydrophilic polymer (PVP): at pH 7.4, the dissolution rate increased with smaller size fractions [44]. Conversely, particle size did not significantly affect the drug dissolution profiles in the case of MPs loaded with 10% *w/w* of PZQ [36].

### 4.3. Shape and Surface Morphology

**Figure 6:**
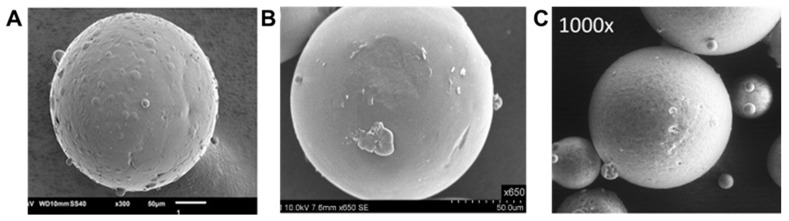
Morphology of spray congealed MPs based on different hydrophilic excipients. (**A**) PEG 3350; (**B**) Poloxamer 407; (**C**) Gelucire 50/13. Figures adapted with permission from [49,50,51].

The shape of MPs is, together with the particle size, another important property influencing both the technological properties of the pharmaceutical formulation (flowability) and the biopharmaceutical performance of the system [46]. SC allows the manufacturing of spherical free-flowing MPs (Figure 6). There is evidence that the most basic functions of particles, such as degradation and release of therapeutic drug, depend on the particle shape [47]. In fact, the particle morphology affected the surface area available for drug transfer in the aqueous solution; moreover, structural defects in the particle matrix might act as preferential paths for drug diffusion. Therefore, MPs characterized by defects on the surface showed faster release rate than smooth microspheres [48]. 

Microscopic analysis of the particle surface can provide important information about the distribution of drug particles, porosity or defects on the particle matrix. Scanning electron microscopy (SEM) is the most commonly used method for the study of MPs shape and surface morphology. SEM analysis uses a monochromatic electron beam to probe the surface and near-surface area of materials at a higher magnification and resolution than a traditional light microscope. Energy dispersive X-ray microanalysis (EDX) is often combined with SEM to provide elemental information about the area probed by the electron beam [52]. Additionally, transmission electron microscopy (TEM), confocal microscopy, and other advanced techniques can be applied for the investigation of the external and internal morphology of the MPs. Recently, synchrotron radiation X-ray micro-computed tomography (SR-μCT), a powerful non-invasive technique, was used for the study of the internal three dimensional structure of spray congealed MPs containing ibuprofen [53]. This method allowed the determination of interior porous channels and irregular structures of the MPs. 

By monitoring the morphology overtime, changing in particle surface appearance might indicate changes in carrier (and/or drug), solid state, or rearrangement of drug/carrier molecules. The surface morphology of Gelucire^®^ 50/13 MPs containing glimepiride was examined using the scanning electron microscopy (SEM) before and after the storage [19]. After 30 days of storage, leaf-like structures were found on the particle surfaces and the change was attributed to the polymorphic transformation of the glyceride portion from α to β form. This phenomenon is known as ”blooming” and has been reported to occur with Gelucire^®^ 50/13 during storage at elevated temperatures [54]. The surface of MPs with the poorly water soluble drug olanzapine prepared by means of ultrasound-assisted spray congealing revealed the presence of wart-like protuberances [55]. The effect was hypothesized to be related to crystallization, phase de-mixing, or lipid component diffusion of the carrier mixture inside the cooling mass subjected to ultrasound vibration, all phenomena that have an important impact on the biopharmaceutical properties and stability of the formulation.

### 4.4. Drug-Carrier Interactions

Interactions between drug and carrier include ionic interaction, hydrogen bond, dipole–dipole interaction, and Van der Waals interaction [56], where hydrogen bond formation is the most common type of interaction observed in SD [57]. Drug-carrier interactions play a critical role in different aspects of the SD and influence their manufacturability, chemico-physical stability, and biopharmaceutical performances.

In a spray congealed systems, the melted carrier and drug particles are intimately mixed prior to atomization. At this stage, interactions between drug and excipient molecules at the liquid state are favored. These interactions are formed during the feed stage, when the carrier is liquid, but they can also persist after solidification of the MPs [56]. The identification of possible interactions between the components at the molten state is particularly important when dealing with spray congealed formulations, because they can influence important properties of the molten mixture, such as its viscosity. Moreover, specific interactions between drug and carrier play a significant role in the formation of eutectic systems [18]. Therefore, specific analysis already in the pre-formulation phase are fundamental. If drug-carrier bonds are formed in the molten mixture, API self-interactions are displaced by drug–carrier interactions, and API crystals lose their ordered tridimensional structure. Conversely, during solidification of the system the pattern is opposite: the intermolecular bonds between API and carrier molecules tend to be displaced by drug self-interactions as well as carrier self-interactions. This tendency results in the progressive crystallization of both components: the API will be excluded from the carrier-rich crystalline phase and, conversely, the drug segregation and crystallization will favor the carrier crystallization. This process has been very clearly explained by Van Duong and co-workers by studying the indomethacin PEG-based SD [58,59]. 

**Figure 7:**
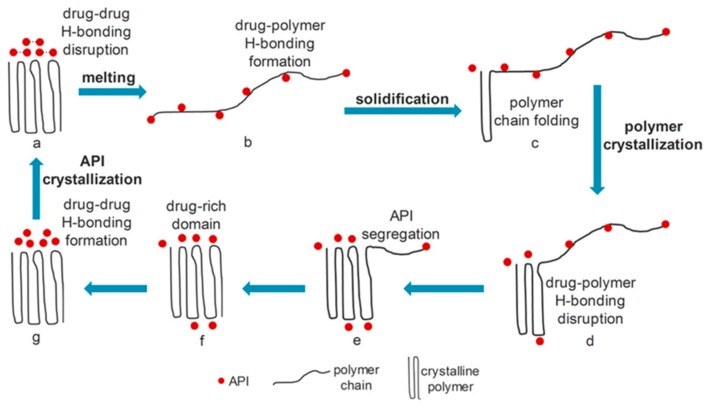
Reprinted with permission from T. Van Duong et al., “Spectroscopic Investigation of the Formation and Disruption of Hydrogen Bonds in Pharmaceutical Semicrystalline Dispersions,” Mol. Pharm., vol. 14, no. 5, pp. 1726–1741, 2017 [59]. Copyright 2017 American Chemical Society.

The scheme illustrated in Figure 7 represents the general behavior of semicrystalline SD, where the progressive carrier and drug crystallization are due to thermodynamic driving forces. However, the rate and extent of this phenomenon are deeply influenced by the strength and nature of drug-carrier interaction as well as by other factors. For example, the formation of metronidazole–PEG interactions in spray congealed SD determined a marked reduction in drug crystallinity (more than 75%) after SC process [49]. The reduction of crystallinity was attributed to drug–PEG interaction as well as to the rapid cooling and solidification of the molten droplets during SC. The rapid solidification of SD hindered the drug molecules from rearrangement into their crystalline form. Strong intermolecular bonds between loaded compound and carrier can provide sufficient energy to maintain the drug in the “unstable” state. Specifically, interactions can help in keeping the drug in the amorphous state or dispersed at a molecular level within the carrier [60]. 

Spectroscopic techniques are the most widely used analysis for the study of interaction mechanism at a molecular level. Fourier-transform infrared (FT-IR) spectroscopy can directly monitor the vibrations of the functional groups that characterize molecular structure at the solid state reactions [61]. It detects changes in the solid state such as hydrogen bonding or π−π interactions which are reflected as peak shifts of functional groups [62]. For example, Kulthe et al. [33] examined the drug polymer interactions between acetazolamide and poloxamer 237 in spray congealed MPs by means of FT-IR. A slight broadening and shift in the position of the free C = O vibration and N–H stretching vibration, characteristic peaks of the drug, to lower wavenumber suggested intermolecular hydrogen bonding in SD. 

### 4.5. Solid State Properties of Drug and Carrier

As previously mentioned, the drug can be present as molecular, amorphous, or crystalline state within the SD, depending on the formulation and preparative process thereof [63]. Extremely important for SD aiming to bioavailability enhancement is the evaluation of the drug solid state, because it deeply affects the drug dissolution performance and therefore, drug bioavailability. In addition, as the carrier normally constitutes the largest part of the formulation, its characteristics greatly contribute to the properties and behavior of the SD. As in the SC technique the solidification from the melt is very fast, this may bring along modifications of the physical form of the carrier in solid state (e.g., transformation from the original crystalline form into a different polymorph). Therefore, the solid state properties of both the drug and the carrier should be carefully evaluated. Moreover, the drug and carrier mutual influence on their specific solid state should be considered. Regarding SD obtained by SC technology, various factors contribute to determine the API solid state: (i) Drug-carrier interaction, as discussed in the previous paragraph; (ii) the nature of the carrier itself, such as its viscosity and crystallization tendency; (iii) the nature of the drug itself; (iv) the drug content; (v) the process parameters, mainly rate and temperature of the cooling phase; and (vi) the storage conditions.

Various techniques can be used to detect the modifications in the solid state of drug and of carrier in the spray congealed systems. Differential scanning calorimetry (DSC) and X-ray powder diffraction (XRPD), as well as variations of these methods are the most used techniques for this purpose. In this context, DSC is mainly employed for the detection of thermal events indicating melting or crystallization processes. Generally, the DSC profile of the drug-loaded MPs is compared with those of the individual raw materials (carrier and API) and/or of their physical mixture. Variations of the drug or carrier melting temperature (e.g., shift at lower temperatures compared to the original drug melting temperature) may indicate a conversion in a different polymorph. Conversely, the disappearance of the drug melting peak could suggest the conversion of the drug in the amorphous form. However, in spray congealed systems based on Gelucire and PEG, the absence (or reduction) of API melting peaks has been noticed even when the drug remained in the crystalline state. This phenomenon has been attributed to a gradual drug dissolution in the melted carrier during the DSC analysis [37,64]. This can cause a lowering and broadening of the drug melting point, as suggested by Craig [65]. Fini et al. reported that during the DSC scanning, the crystalline drug diclofenac dissolved into the molten PEG 6000 starting from 60 °C [66]. In case of SD of Gelucire 50/13 and PEG 4000, the drug indomethacin showed only a weak broad peak shifted to lower melting point, and similar profiles were obtained from the physical mixture [67]. The same phenomenon has been noticed for Gelucire 53/10 and Poloxamer 188-based MPs containing econazole nitrate [68] as well as for Gelucire 50/13-based MPs containing Silybum Marianum dry extract [69]. It is important to consider that the extent of this phenomenon is related to the relative amount of drug and carrier in the formulation. For example, the melting endotherm of carbamazepine, loaded from 5 to 20% *w/w* into Gelucire 50/13 MPs, was not detected in DSC analysis [37]. The melting peak of PZQ contained in spray congealed MPs of Gelucire 50/13 was observed in the DSC profile when the drug content was as high as 30% *w/w* [36] while in another study, the same API loaded at 15% *w/w*, completely solubilized in the molten Gelucire 50/13 during the scan [70]. Specifically, in case of high drug amounts, the amount of molten carrier is not sufficient to dissolve the drug crystals during DSC scan. Thus, the melting of the undissolved crystalline drug appears when the amount of drug exceeded its solubility in the molten carrier. 

DSC data are important to obtain information on the solid state of the low melting point carriers; however they are often not sufficient to establish the physical state of the drug in the SD and thus other techniques should be employed to investigate this aspect. Hot stage microscopy (HSM) analysis is based on the observation of the samples by means of a microscope while they are heated in a furnace in which the heating or cooling rate can be accurately controlled. Although HSM is a qualitative evaluation, the presence of crystals after carrier melting is generally easy to observe, even at small magnifications. HSM is complementary to DSC and particularly useful to interpret or to confirm the DSC thermal profiles, especially when the thermograms present overlapping events. Another technique for the study of the solid state properties is polarized light microscopy (PLM). PLM exploits the optical properties of crystalline solids by using plane polarized light or crossed polarizers. This is possible because most crystalline solids are anisotropic, which means their molecules are packed in a regular, long-range, three-dimensional order and exhibit interference colors or birefringence when observed between crossed polarizers [52]. To allow a better observation of the crystal forms, HSM images can be taken under polarizing light, a method defined as hot-stage polarized light microscopy (HSPLM). 

Finally, to gather more information on the drug solid state properties after SC process, very useful is the XRPD analysis, which provides information on the crystalline phase of the sample by the presence of diffraction reflections [71]. The comparison of diffractograms of drug-loaded MPs with their physical mixtures provide a clear indication about the solid state properties of the API: if the two diffractograms have analogous peak patterns with comparable intensity of the peaks, then the occurrence of drug modifications during the SC process can be excluded. In particular, XPRD can provide unquestionable information about the maintenance of original drug polymorph (peaks at the same diffraction angles), formation of a different polymorph (reflections at different angles), amorphization (absence of sharp diffraction peaks, called “amorphous halo”), and crystallinity reduction (less sharp and less intense diffraction peaks).

For formulations at low drug contents (≤10%), the levels of crystallinity may approach the limits of detection of the common characterization methods such as DSC and PXRD (commonly ∼1–5% by mass). In this case, solid-state NMR can achieve an order of magnitude of lower detection limits (∼0.1%), even though the drawback of long measurement times is present [72].

### 4.6. In Vitro Evaluation of the Biopharmaceutical Properties

In vitro methods have been developed to evaluate the release as well as the permeation of the API after oral administration. The release profile of the API from the MPs is generally determined by in vitro dissolution studies, carried out according to Pharmacopoeia dissolution apparatus, such as apparatus I, II, and IV [73], although non-official apparatus are also often used. The amount of drug released is assayed by an appropriate analytical method. Generally, the dissolution tests employ “simple” experimental conditions (e.g., sink conditions using a single well-defined medium and volume at a constant pH). These conditions are quite different from the in vivo situation, where GI transit exposes the drug/formulation to a rapidly changing and complex luminal environment [74]. To better predict the in vivo behavior, media simulating the GI fluids for the in vitro dissolution tests were developed [75]. They are called biorelevant media and closely mimic the GIT environments in both fasted (without food) and fed (with food) states. Moreover, it should be considered that poorly water-soluble drugs may precipitate in vivo during the transit through the GIT. Changes in local environmental pH, dilution of the formulation with body fluids, or digestion of solubilizing excipients which compose the formulation are possible reasons for drug precipitation. 

However, the effects governing the dissolution rate from SD are not completely explained [76]. The reason for this lies in the complexity of the mechanism of API release from SD and the multitude of factors that can influence it [77]. Back in 2002, Craig [65] identified two different types of drug release from soluble carrier-based SD namely carrier-controlled and drug-controlled dissolution, the predominance depending on the solubility of the drug in concentrated solutions of the carrier. In the former type, the particles dissolve first into the polymer-rich diffusion layer on the particle external surface and later into the aqueous medium. In this case, the drug solubility in the carrier is so high that the direct release into the medium is prevented. In the latter type, the drug is released effectively intact into the dissolution medium. Here, the dissolution is not dependent on the carrier but is determined by the properties (size, physical form, etc.,) of the drug itself. Nevertheless, the understanding of the dissolution from SD remains difficult, as multiple phenomena such as supersaturation and API crystallization are involved and can affect the dissolution behavior [76].

As regard the assessment of drug absorption of the API upon release from the MPs, cell models simulating the intestinal membrane are employed to test the amount of drug permeability. Those cell models are extensively used as they represent an easy and reproducible method, allowing the tracking of drug absorption rate and mechanism, with an advantageous cost–benefit ratio [78]. They are mainly composed of immortalized cells with the ability to grow in permeable supports forming a confluent monolayer, maintaining their physiologic characteristics regarding epithelium cell physiology and functionality (e.g., microvilli, tight junctions). The most diffused in vitro models for permeability studies is the Caco-2 model, a human epithelial colorectal adenocarcinoma cell line. Typically, Caco-2 cells are cultured for ∼21 days on porous filter supports until formation of a fully differentiated and polarized monolayer [79]. A number of alternative in vitro models have also been employed in permeability studies of poorly soluble drugs, including co-culture systems [80] as well as non-cellular models [81,82].

Finally, it is important to consider that the dissolution of the API from the SD and its absorption are intimately connected. For example, the absorption of BCS class II drugs, which possess high permeability, creates a sink effect that may increase dissolution and decrease precipitation. Also, if most drugs are incorporated in micelles derived from the bile acids of the biorelevant media, the rate of oral absorption may be affected [83]. Therefore, to take into account this dissolution-permeability interplay, methods based on the assessment of permeability from relevant samples, i.e., originating from solubility or dissolution experiments, have been developed [84,85]. An integrated dissolution/Caco-2 system was developed combining two in vitro technologies: dissolution testing and Caco-2 monolayers. This allowed a more accurate prediction of the contributions of dissolution and permeation to the overall drug absorption performance [84].

## 5. Mechanisms of Bioavailability Enhancement of Spray Congealed Systems

A number of spray congealed systems has been developed for the bioavailability enhancement of different poorly soluble drugs. These formulations can be divided in two categories:

- MPs containing the drug used *as received*
- MPs containing *pre-activated* drug

As regard the first group, spray congealed SD of different APIs and excipient have been explored over the years (Table 1). The mechanisms of bioavailability enhancement of these systems are largely dictated by the structure of the SD formed after the SC process (see Section 3). Specifically, the increase in oral bioavailability by spray congealed SD depends on multiple effects, which can be divided in two main categories: (i) Effects that are independent on the API physical state inside the MPs and (ii) the effects resulting from the changes in the drug crystalline original state. Whereas the second category is strictly dependent on the specific API/carrier combination should be therefore studied on a case-by-case basis, the first one can be ideally exploited for the bioavailability enhancement of all poorly water soluble drugs.

When the original drug solid state is maintained after the process, the possible mechanisms of bioavailability enhancement consist in (i) improved wetting, (ii) enhanced solubilization, iii) formation of dispersed systems and iv) reduction of drug particle size. First, hydrophilic excipients increase API wettability as they act as wetting agents by favoring the contact between hydrophobic drug particles and water. Moreover, when surface active carriers are utilized (e.g., Gelucires^®^), a dispersed system (e.g., O/W emulsion or micellar dispersion) can be spontaneously formed upon aqueous dilution with the GI fluids. For example, spray congealed MPs containing glibenclamide showed self-emulsifying properties [86]. In this regard, formulations based on Gelucire 50/13 showed good in vitro dissolution enhancements because of the formation of a dispersed phase. The addition of a co-solvent (PEG400) or co-solvent and surfactant (PEG 400 and Poloxamer 188), further improved the drug solubilization rate. These formulations formed a colloidal system in aqueous medium (at 37 °C) within 60 minutes with an average micelle size of ca. 350 nm. In another study employing glimepiride, Gelucire^®^ 50/13 was the carrier showing the fastest dissolution rate, compared to Poloxamer 188 and PEG 6000 [19]. Additionally, the drug solubility was increased by about 10-fold using Gelucire and about 5-fold by using Poloxamer 188 and PEG 6000. It has also been suggested that the encapsulation of drug particles into MPs as physically separate entities may reduce the aggregation and agglomeration during release. Qi et al. suggested that during dissolution, Gelucire^®^ 50/13-based microspheres are subjected to swelling (hydration) and formation of a liquid crystalline phase. This process can facilitate the wetting of the drug particles embedded in the microspheres and maximize the surface area via prevention of aggregation, high surface area, and well-wetted state [87]. Reduction in drug particle size after SC process is another mechanism for improved dissolution rate. Ultrasound-assisted SC was employed to produce MPs of Gelucire^®^ 50/13 and various amounts (9, 18, 36 and 45% *w/w*) of Lutrol F68 and F127 containing 10% *w/w* of olanzapine [55]. Olanzapine dissolved into the molten carrier, but solidified in reduced-sized crystals. This effect led to an accelerated release (90% of drug released in the first 10 min) with respect to the pure active ingredient (only 15% in the first 10 min). 

In addition to the above mentioned mechanisms, the increase in dissolution rate of spray congealed SD can be due to modifications of the API original crystalline form. The formation of a one-phase liquid mixture during the first step of the SC process because of solubilization of the API into the molten carrier is a necessary, but not a sufficient condition for the occurrence of a solid state modification of the API. If the API interactions with the molten carrier are strong enough to allow the solubilization of the drug, the resulting MPs may present reduced drug crystallinity with partial or total conversion into the amorphous form. In this case, during cooling, the solubilized drug re-solidify in disordered clusters within the carrier matrix, with a complete or partial loss of the ordered crystalline structure. Reduced drug crystallinity has been reported for spray congealed MPs containing metronidazole [49] and diclofenac [88], whereas complete conversion into the amorphous form was observed for acetazolamide [33]. It is well-known that solids in the amorphous state have a higher solubility, as no energy is required to break up the crystal lattice in the dissolution process. In the cooling phase, the solubilized drug may also crystallize into a different polymorph, which is usually a metastable form, characterized by a higher energy compared to the thermodynamic stable form. The BCS class II drug carbamazepine (CBZ) has been formulated into spray congealed SD based on Gelucire^®^ 50/13 [37,64]. In both cases, the Gelucire^®^-based SD seemed to stabilize CBZ in the metastable α polymorph. Whereas in the study from Passerini et al. [37] the original CBZ was a mixture of polymorphs β and α, the study of Martins et al. [64], which employed CBZ entirely in the stable β-form, clearly showed the conversion of the drug into the metastable α-form. Finally, SC may lead to the formation of a molecular dispersion of drug within the carrier. In a recent study from Zuo et al. [50], poloxamer 407 was employed for the production of MPs containing three compound of the class of bufadienolides, leading to a four-fold increase in vitro dissolution rate because of the formation of a system with molecular dispersed drug. However, whereas all these studies confirmed the efficacy of spray congealed SD in increasing drug dissolution in vitro, very few studies tested these formulations using in vivo models. A recent work from our group [45] demonstrated that the positive in vitro results in terms of solubility and dissolution enhancement of spray congealed Gelucire^®^-based SD of the poorly water soluble drug indomethacin were also confirmed in vivo on rats. In fact, after oral administration of MPs, the drug maximum blood concentration increased from 10 µg/mL to about 24 µg/mL and the absolute bioavailability was enhanced by 2.5-times. Notably, the absolute bioavailability of the original crystalline form of the drug, equal to 19.2% , increased to only 21.0% by administering a physical mixture of drug and carrier, but it was enhanced up to 47.5% in case of spray congealed formulation. This result clearly indicates that the effects related to the presence of the hydrophilic carrier were not sufficient for determining a bioavailability enhancement in vivo, whereas the improved pharmaceutical outcome observed in case of spray congealed SD suggest that the mechanisms involved in the bioavailability enhancement are dependent on the SC process.

A less used but very interesting strategy involves the incorporation of pre-activated drug into the MPs. Instead of loading the MPs with commercial drug powder, different treatments causing physical and/or chemical transformations of the original solid powder can be used prior to SC process to produce pharmaceutical solids in an extremely "active" state. For example, Fini et al. [90] prepared a primary SD of indomethacin and polyvinylpyrrolidone (PVP) by dissolving both in a common solvent (ethanol) following by solvent evaporation. The obtained SD, defined as co-evaporated, consisted on an amorphous system which was then processed by SC. Stearic acid was selected as the carrier for achieving gastroprotection as well as stabilization of the amorphous form of the drug. SC allowed protection of co-evaporation from the external environment, improved the technological properties of the system and prevented drug re-crystallization for at least 9 months. Another approach involves the mechanochemical activation of the API, based on processing the solid drug in a mill where mechanical energy is applied and transferred on the solid material surfaces. It represents a solvent-free process able to ameliorate the biopharmaceutical properties (e.g., solubility, intrinsic dissolution rate) of APIs due to the formation of different activated physical forms (e.g., amorphous/nanocrystalline or polymorphous) [91]. However, milled drug powders often present poor technological properties in term of flowability and thus, processability into solid dosage forms. The association of mechanochemical activation with SC technology has the potential to improve the technological properties of the drug powder by loading the grounded system into spherical free-flowing MPs, resulting in a better product handling. Moreover, the encapsulation into hydrophilic MPs may further improve the bioavailability of the poorly soluble drug. The combination of these two techniques was applied for the first time to Silybum Marianum dry extract, which was first co-ground with sodium croscarmellose in a planetary mill, resulting in nanosized particles of dry extract with reduced degree of crystallinity [69]; the co-ground extract was then loaded in Gelucire-based MPs prepared by SC technology. The systems obtained combining the two techniques showed a favorable in vitro solubilization kinetic. In addition, a preliminary in vivo study in five rats demonstrated that the system substantially improved the oral bioavailability of the extract main components. Recently, a similar approach was used to develop a child-friendly dosage form of PZQ, the first line drug used in endemic countries for the treatment and prevention of schistosome infections [70]. The high dose required for PZQ to be effective per os administration, because of its low aqueous solubility and high first pass effect, make this drug available in large tablets difficult to be swallowed by children [11]. To overcome this problem, activated materials of PZQ were prepared by grounding the crystalline drug by itself to form a metastable polymorph (form B) or together with PVP at low temperatures (cryo-co-grounding) and were subsequently loaded into the MPs by SC, using Gelucire^®^ 50/13 as carrier [70]. After the SC process, the results evidenced that the cryo-co-ground and the milled PZQ formed either a solid dispersion (nanocrystalline and partial amorphous phase) or a solid solution (completely amorphous state), respectively. The final MPs showed a further increase in solubility and a marked improvement in the rate of dissolution compared to the milled powders.

**Figure 8:**
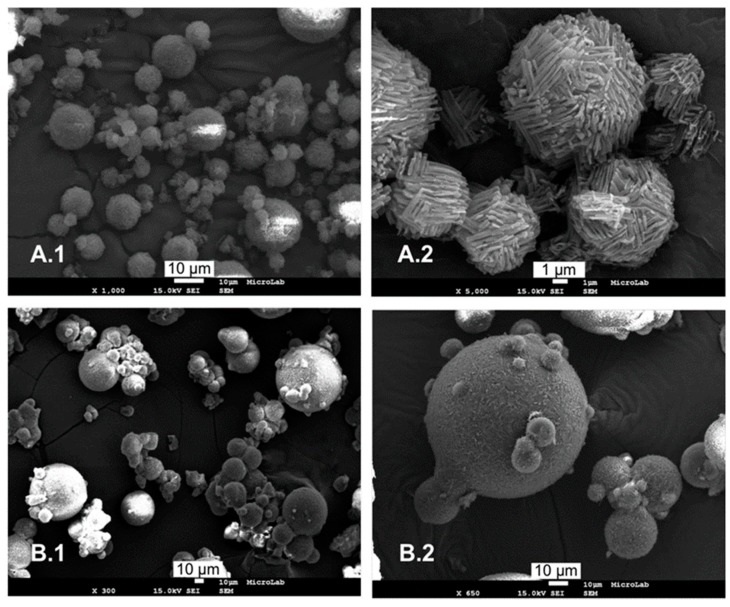
Micrographs correspondent to the cocrystals obtained by spray congealing with caffeine (**A**) and carbamazepine (**B**). Reprinted with permission from [92].

Finally, it is worth mentioning a recent innovative application of SC for the bioavailability enhancement of poorly soluble drugs, developed by Duaerte and coworkers [92]. In spite of a SD of drug and carrier, SC has been used to produce co-crystals in the form of MPs. Pharmaceutical co-crystals are stable multicomponent crystals, typically comprising the API (or the salt of the API), and a crystals former or co-former. The co-former can be either an active or non-active ingredient (e.g., vitamins, minerals, amino acids, other APIs or an inert pharmaceutical excipient) able to form a stable intermolecular interaction with the API, including van der Waals contact forces, π⋯π stacking interactions, and hydrogen bonding [93]. Pharmaceutical co-crystals have the potential to improve desired pharmaceutical properties, including solubility and dissolution behavior, without modifying the chemical structure of drug molecule and thus maintaining its therapeutic activity [94]. SC appears as a green (solvent-free) alternative method for the production of pharmaceutical co-crystals. Co-crystallization via SC complies with green chemistry and sustainable pharmacy principles, allows cost reduction and avoids the formation of solvates. In this innovative application of SC technology, the model APIs (caffeine and carbamazepine) and respective conformers were heated up until melting of the mixture prior to atomization. The spray congealed co-crystals were spherical-shaped (Figure 8) with size around 4–6 μm. The thermal analysis and PXRD analysis of the spray-congealed co-crystals confirmed substantial differences from the pure components or physical mixtures and showed all the properties of the same co-crystal systems produced by other methods.

## 6. Conclusions and Future Perspectives

The production of feasible oral formulations of poorly soluble drugs remains a challenge for the pharmaceutical industry. SC is an emerging technology within academia, which could allow the industrial production of SD for the bioavailability enhancement of poorly soluble drugs by using low-melting hydrophilic excipients. The main advantages are the absence of solvents and the possibility to obtain spherical free-flowing MPs with a cheap, simple, one-step process. To date, the majority of spray congealed SD for bioavailability enhancement of poorly water soluble drugs have directly employed the commercial API as received, while few applications employed pre-activated drugs. One of the most interesting recent development of this technology consists in the application of SC for the production of cocrystals. 

To date, the methods for the characterization of the solid state and physico-chemical properties of the spray congealed systems are well-established. A number of case studies in the literature have showed that spray congealed SD have the potential to improve the drug bioavailability of poorly soluble drug by different mechanisms, such as increased wettability, dispersed system formation, size reduction of API crystals, conversion of the API into metastable polymorphs or into the amorphous form. However, the contribution of the different mechanisms to the overall biopharmaceutical performance of the spray congealed MPs has not been entirely clarified. Moreover, the bioavailability enhancement of these systems has been demonstrated mainly by in vitro tests, whereas only very few in vivo studies on animal models are available in the literature. Thus, the future research in this field should further investigate the underlying mechanisms of increased dissolution and solubility of SD produced by SC. Additionally, more in vivo data are required to confirm the positive in vitro results.

## Figures and Tables

**Table 1:** Application of spray congealing to oral bioavailability enhancement of poorly water soluble drugs used without pre-activation.

Drug	Carrier + Additives	Type of SD	Achievement	Ref.
Carbamazepine	Gelucire^®^ 50/13	Crystalline drug (original polymorph)	Increased in vitro dissolution rate	[37]
Carbamazepine	Gelucire^®^ 50/13	Crystalline drug (change from β to α)	Increased in vitro dissolution rate	[64]
Piroxicam	Gelucire^®^ 50/13	Crystalline drug (original polymorph)	Increased in vitro dissolution rate	[87]
Praziquantel	Gelucire^®^ 50/13	Crystalline drug (original polymorph)	Increased in vitro dissolution rate	[36]
Olanzapine	Gelucire^®^ 50/13, Lutrol F68 or Lutrol F127	Reduced-sized drug particles	Increased in vitro dissolution rate	[55]
Acetazolamide	Poloxamer-237	Conversion to amorphous form/ dispersion on molecular level	Increased in vitro dissolution rate and 9-fold solubility enhancement	[33]
Glibenclamide	Myverol, Myvatex, Gelucire^®^ 50/13, Gelucire^®^ 44/14, Poloxamer 188 and PEG 4000	Crystalline drug	Five-fold increased drug solubilization (Gelucire 50/13 + PEG 4000)	[86]
Metronidazole	PEG 3350 + HPMC, Dicalcium phosphate, Magnesium stearate, Methylcellulose, Polyvinylpyrrolidone, Silicon dioxide, Sodium oleate/Citric acid	Marked reduction in drug crystallinity	Slightly increased in vitro dissolution rate with only carrier (different results depending on the type and amount of additive)	[49]
Rifampicin	PEG 3350 + HPMC (different grades)	No information provided	Increased in vitro dissolution rate, with intensity depending on the HPMC grade.	[89]
Glimepiride	Gelucire^®^ 50/13, poloxamer 188, and PEG 6000	Crystalline drug (original polymorph)	Increased in vitro dissolution rate (Gelucire the highest increase). 10-fold (Gelucire) and 5-fold (poloxamer, PEG) solubility enhancement	[19]
Diclofenac	Gelucire^®^ 50/13	Marked reduction in drug crystallinity	Increase in the *in vitro* dissolution rate	[88]
Indomethacin	Gelucire^®^ 50/13, Gelucire^®^ 48/16	Conversion to amorphous form/dispersion on molecular level	Increase in the in vitro dissolution rate and solubility Increased oral bioavailability in rats	[45]
Bufadienolides (bufalin, cinobufagin, and resibufogenin)	Lutrol F127	Formation of molecular dispersions	Four-fold increase in vitro dissolution rate	[50]
Wild garlic extract	Gelucire^®^ 50/13	*No information provided*	Increase in the in vitro dissolution rate	[51]
